# A silent storm

**DOI:** 10.1002/rcr2.35

**Published:** 2013-12-10

**Authors:** Chiow Teen Lim, Lee Lan Phoa

**Affiliations:** Department of Respiratory and Critical Care Medicine, Khoo Teck Puat HospitalSingapore, Singapore

**Keywords:** Pulmonary alveolar microlithiasis, pulmonary calcifications, pulmonary fibrosis

## Abstract

Pulmonary alveolar microlithiasis is a rare, inherited pulmonary disorder affecting young adults. Diagnosis and monitoring is important as it may progress to pulmonary fibrosis and respiratory failure. No effective treatment has been found to date.

## Introduction

Pulmonary alveolar microlithiasis (PAM) is a rare disorder first described in the 17th century. There are currently less than 1000 cases reported in the literature worldwide. We report a case of PAM in a young male patient with an incidental abnormal chest radiograph during his preoperative assessment for acute appendicitis. He has no respiratory symptoms and has normal lung function despite radiological and histopathological features of PAM. He had an uneventful surgery under general anesthesia with good recovery. There is currently no proven treatment for PAM. Patients with PAM are at risk of developing pulmonary fibrosis with cor pulmonale and progressive respiratory failure. Lung transplant may then be a viable option.

## Case Report

**Figure 1:**
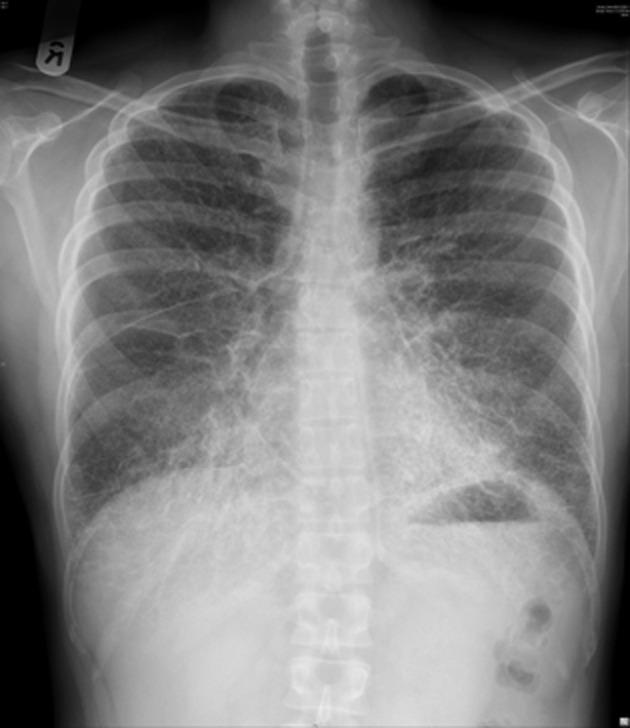
Plain chest radiograph showing a typical “sandstorm” appearance of pulmonary alveolar microlithiasis.

**Figure 2:**
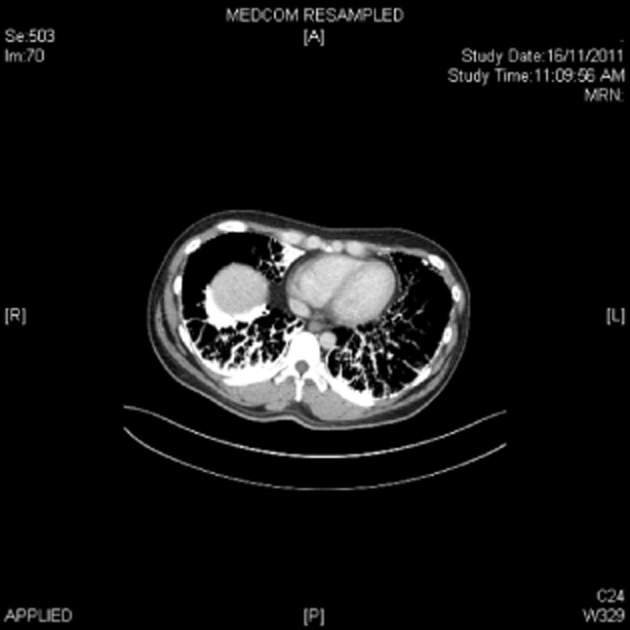
Computed tomograph in the bone window showing typical calcifications due to accumulations of microliths in the alveoli.

**Figure 3:**
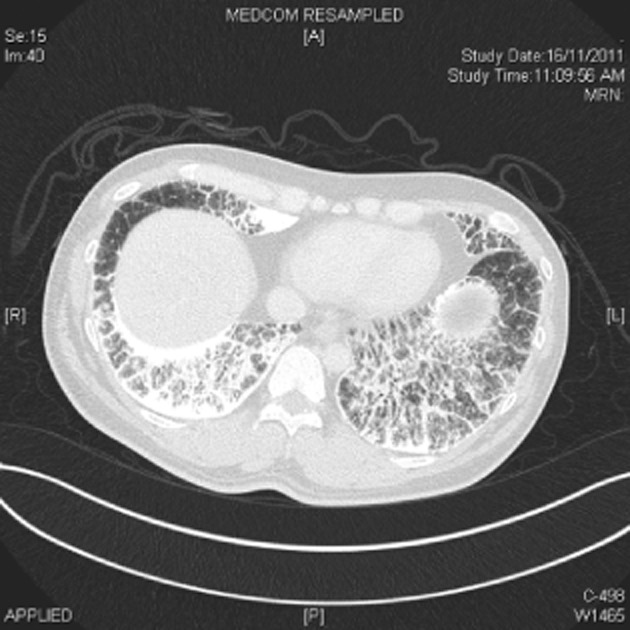
High-resolution computed tomography of thorax in lung windows showing septal thickening associated with pulmonary alveolar microlithiasis.

A 38-year-old man was admitted with a diagnosis of acute appendicitis via the Emergency Department. Preoperative assessment chest radiograph (Fig. 1) was found to be abnormal and a computed radiograph (CT) of the thorax was performed (Figs. 2 and 3).

The patient had no respiratory symptoms prior to his current admission for acute appendicitis. He has no past medical history of note in particular that of childhood respiratory illnesses, tuberculosis, renal, or metabolic disorders. There is no significant family history of any medical illness except that his male sibling reportedly had an abnormal chest radiograph but refused investigation. He works as a full-time clerk and is married with two children. He does not keep any pets at home. He has no travel history. He smokes approximately 15 cigarettes daily for the last 20 years and does not drink alcohol.

He was comfortable at rest with no requirement for supplemental oxygen. His oxygen saturation remained above 97% on room air. There were no abnormal findings on physical examination, except for tenderness over the right iliac fossa.

Blood investigations were unremarkable except for an elevated white cell count with neutrophilic predominance in keeping with acute appendicitis. CT thorax however showed diffused interlobular and intralobular septal thickening, suggesting interstitial calcifications and innumerable tiny nodular opacities, predominantly at the lung bases. There was also bilateral subpleural emphysema seen in both upper lobes in keeping with a smoking history. The abdominal CT did not reveal any extrapulmonary calcification.

The patient underwent appendicectomy under general anesthesia without complications and had an uneventful recovery.

**Figure 4:**
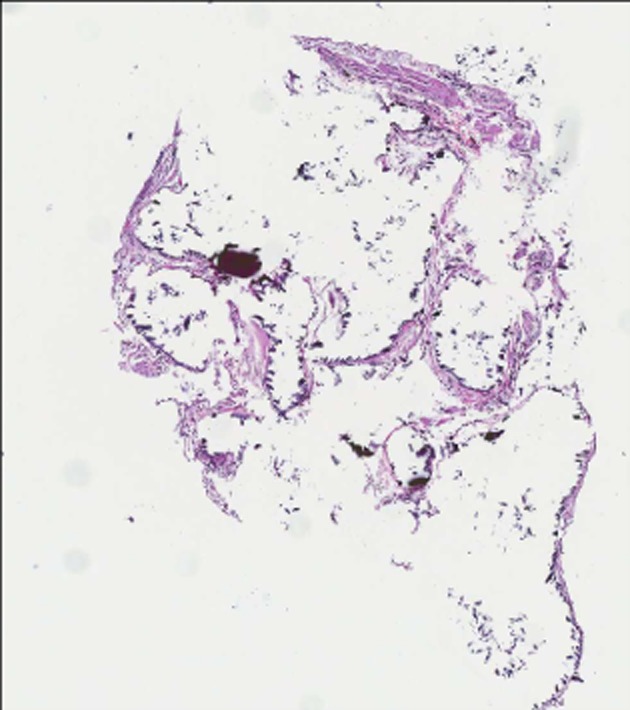
High power view showing hematoxylin and eosin stained alveoli with calcified structures (black dots) within but separated from each other by thin alveolar septa.

Bronchoscopy with transbronchial lung biopsy was subsequently performed. Histopathological examination of the biopsy specimens confirmed the diagnosis of PAM (Fig. 4). Spirometry, lung volumes, and diffusion test were performed during his follow-up visit. The results of the pulmonary function tests are within acceptable limits except for a marginally reduced DLCO/VA (rate of carbon monoxide uptake) value (see Table 1). The patient was informed of his diagnosis of PAM and the need for long-term follow-up to monitor his respiratory function. He has been reviewed annually in the specialist outpatient clinic with repeat pulmonary function test each year. Smoking cessation advice has been given on each visit. The risk of disease progression causing pulmonary fibrosis and respiratory impairment has been explained to him and he will be referred for consideration of transplant should this occur. His male sibling with the reportedly abnormal chest radiograph was offered an evaluation but he refused further investigation.

**Table 1:** Lung function test showing marginal reduction in DLCO

Spirometry	Pre	% predicted
FEV_1_ (L)	2.98	97
FVC (L)	3.89	108
FEV_1_/FVC (%)	76.7	
Lung volume – He		
RV (L)	0.97	60
TLC (L)	5.07	86
VC (L)	4.10	81
Diffusion test		
DLCO (mmol/min/kPa)	6.67	71
DLCO/VA (mmol/min/kPa/L)	1.21	65

DLCO, carbon monoxide diffusing capacity; FEV_1_, forced expiratory volume in 1 sec; FVC, forced vital capacity; RV, residual volume; TLC, total lung capacity; VA, alveolar volume; VC, vital capacity.

## Discussion

PAM is a rare genetic disorder first described in the 17th century based on autopsy findings [1]. However, more accurate description and case series was not reported until the 20th century. There are still less than 1000 cases reported in the literature, in both adult and children worldwide to date [1, 2]. It is a disorder with male predominance, and both familial as well as sporadic cases have been reported. The highest number of cases reported so far was from Europe closely followed by Asia.

PAM is characterized by the finding of innumerable microliths or small calculi made predominantly of calcium phosphate deposition in the alveolar space. This may lead to progressive ventilation perfusion mismatch, diffusion impairment, respiratory failure, pulmonary hypertension, and cor pulmonale [1]. Extrapulmonary involvement has been described in the literature, such as pleural, cardiac, or urogenital tract involvement [3, 4].

The specific genetic mutation responsible for causing PAM has now been identified as the *SLC34A2* gene, which encodes a type IIb sodium-dependent phosphate transporter highly expressed in the type II alveolar cells [5]. Type II alveolar cells are responsible for production, recycling, and degradation of pulmonary surfactant. Degraded pulmonary surfactant, consisting mainly of phospholipids, releases phosphate, which failed to be cleared efficiently with *SLC34A2* mutation. The reduced clearance of phosphate leads to the formation of microliths and PAM. PAM is inherited as an autosomal recessive disorder and is therefore prevalent in communities with a high rate of consanguinity among parents of affected individuals [5].

PAM is characterized by diffused calcific micronodules on chest X-ray and CT, giving a “sandstorm-like” appearance [1]. This is more marked on the middle and lower zones, and high-resolution images may reveal parenchymal abnormalities associated with PAM. The diagnosis of PAM can be confirmed by bronchoscopic examination and transbronchial lung biopsy, which is expected to show parenchymal and alveolar calculi and calcification.

There is no proven treatment for PAM to date. Long-term follow-up of patients with PAM is recommended in view of the potential sequelae. Lung transplantation remains the only viable option for patients who suffer progressive respiratory failure secondary to PAM [1].

## References

[1] Castellana G, Lamorgese V (2003). Pulmonary alveolar microlithiasis. World cases and review of the literature. Respiration.

[2] Ucan ES, Keyf AI, Aydilek R (1993). Pulmonary alveolar microlithiasis: review of Turkish reports. Thorax.

[3] Kacmaz F, Alyan O, Celenk M (2007). A case of pulmonary alveolar microlithiasis with cardiac constriction secondary to severe adjacent pleural involvement. Cardiology.

[4] Giallauria F, Giallauria G (2011). Pulmonary alveolar microlithiasis. Thorax.

[5] Huqun, Izumi S, Miyazawa H (2007). Mutations in the SLC34A2 gene are associated with pulmonary alveolar microlithiasis. Am. J. Respir. Crit. Care Med.

